# The influence of time on the sensitivity of SARS-CoV-2 serological testing

**DOI:** 10.1038/s41598-022-14351-2

**Published:** 2022-06-22

**Authors:** Arturo Torres Ortiz, Fernanda Fenn Torrente, Adam Twigg, James Hatcher, Anja Saso, Tanya Lam, Marina Johnson, Helen Wagstaffe, Rishi Dhillon, Anabelle Lea Mai, David Goldblatt, Rachel Still, Matthew Buckland, Kimberly Gilmour, Louis Grandjean

**Affiliations:** 1 Department of Infection, Inflammation and Immunity, Great Ormond Street Institute of Child Health, University College London, 30 Guilford Street, London, WC1N 1EH UK; 2 Department of Infectious Diseases, Imperial College London, Paddington, London, W2 1NY UK; 3 UCL Medical School, University College London, 74 Huntley Street, London, WC1E 6DE UK; 4 Department of Infectious Diseases, Great Ormond Street Hospital, Great Ormond Street, London, WC1N 3JH UK; 5 School of Clinical Medicine, University of Cambridge, Cambridge Biomedical Campus, Box 111, Cambridge, CB2 0SP UK; 6 Department of Microbiology, Great Ormond Street Hospital, Great Ormond Street, London, WC1N 3JH UK; 7 Department of Tropical and Infectious Diseases, LSHTM, Keppel St, Bloomsbury, London, WC1E 7HT UK; 8 MRC Gambia at LSHTM, PO Box 273, Fajara, The Gambia; 9 Public Health Wales Microbiology, University Hospital of Wales, Heath Park Way, Cardiff, CF14 4XW UK; 10 Clinical Immunology, Camelia Botnar Laboratories, Great Ormond Street Hospital, Great Ormond Street, London, WC1N 3JH UK; 11 Laboratory Medicine Service Swansea, Bay University Health Board Morriston Hospital, Swansea, SA6 6NL UK

**Keywords:** Infectious diseases, Viral infection

## Abstract

Sensitive serological testing is essential to estimate the proportion of the population exposed or infected with SARS-CoV-2, to guide booster vaccination and to select patients for treatment with anti-SARS-CoV-2 antibodies. The performance of serological tests is usually evaluated at 14–21 days post infection. This approach fails to take account of the important effect of time on test performance after infection or exposure has occurred. We performed parallel serological testing using 4 widely used assays (a multiplexed SARS-CoV-2 Nucleoprotein (N), Spike (S) and Receptor Binding Domain assay from Meso Scale Discovery (MSD), the Roche Elecsys-Nucleoprotein (Roche-N) and Spike (Roche-S) assays and the Abbott Nucleoprotein assay (Abbott-N) on serial positive monthly samples collected as part of the Co-STARs study (www.clinicaltrials.gov, NCT04380896) up to 200 days following infection. Our findings demonstrate the considerable effect of time since symptom onset on the diagnostic sensitivity of different assays. Using a time-to-event analysis, we demonstrated that 50% of the Abbott nucleoprotein assays will give a negative result after 175 days (median survival time 95% CI 168–185 days), compared to the better performance over time of the Roche Elecsys nucleoprotein assay (93% survival probability at 200 days, 95% CI 88–97%). Assays targeting the spike protein showed a lower decline over the follow-up period, both for the MSD spike assay (97% survival probability at 200 days, 95% CI 95–99%) and the Roche Elecsys spike assay (95% survival probability at 200 days, 95% CI 93–97%). The best performing quantitative Roche Elecsys Spike assay showed no evidence of waning Spike antibody titers over the 200-day time course of the study. We have shown that compared to other assays evaluated, the Abbott-N assay fails to detect SARS-CoV-2 antibodies as time passes since infection. In contrast the Roche Elecsys Spike Assay and the MSD assay maintained a high sensitivity for the 200-day duration of the study. These limitations of the Abbott assay should be considered when quantifying the immune correlates of protection or the need for SARS-CoV-2 antibody therapy. The high levels of maintained detectable neutralizing spike antibody titers identified by the quantitative Roche Elecsys assay is encouraging and provides further evidence in support of long-lasting SARS-CoV-2 protection following natural infection.

## Introduction

Following natural infection or vaccination, sensitive measurement of SARS-CoV-2 serological status is important to identify immune correlates of protection from future waves of the pandemic, evaluate those in need of booster vaccination and identify candidates for SARS-CoV-2 antibody therapy. The rapid response to the COVID-19 pandemic has led to the development of a wide range of serological tests suitable for evaluating SARS-CoV-2 exposure, infection or vaccination status^1–3^. Typically, these tests are approved for use by the regulatory authorities based on their performance against a panel of reference sera including positive and negative controls at either 14- or 21-days post infection^4^.

Public Health England reported a 93.9% sensitivity for the Abbott SARS-CoV-2 IgG Nucleoprotein assay^5^ and 100% for the Roche Elecsys Nucleoprotein assay at ≥ 14 days post infection^6^. This led to widespread adoption of these tests across NHS laboratories for testing at population level. Other studies have confirmed this test performance at 14–21 days post infection^7,8^. Population level serological studies have also based their conclusions—vital to guide national policy—on the basis of these tests^9^ without considering how time since infection influences the performance of the test. The problem with this approach is that it does not take into account SARS-CoV-2 humoral dynamics and changes in avidity over time^10,11^. Although serological tests with limited diagnostic range may demonstrate excellent sensitivity shortly after infection, it is unclear how they will perform with time following infection or vaccination.

In order to address this question, we applied 4 widely used serological assays in parallel to serial samples from the Co-STARs study^12^ in which staff testing seropositive to SARS-CoV-2 were followed for up to 200 days following infection. We compared the proportion of samples that remained seropositive over time using a survival analysis and determined the decay rate of the nucleoprotein (N) antibody and the spike (S) antibody for each test using a previously published mathematical model fitted to the data.

## Materials and methods

### Study setting and design

Serological testing was performed on stored serum samples collected as part of the Co-STARs study (www.clinicaltrials.gov, NCT04380896), approved by the UK National Health Service Health Research Authority and run at Great Ormond Street Hospital between April 29th and November 2020 in accordance with the relevant guidelines^10^. Briefly, Co-STARs was a 1-year single-centre prospective cohort study of antibody responses to COVID-19 infection in healthcare workers. Serum samples were taken from the 3657 participants at baseline and underwent a screening ELISA using the EDI assay. Repeated monthly serum samples were then taken from those with a seropositive baseline screening test for up to 250 days after the date of infection. Written informed consent was obtained from all participants. Those samples identified as seropositive with available symptom start date had further confirmatory testing with the quantitative three antigen MSD assay.

### Study participants

The majority of hospital staff were eligible for the Co-STARS study^12^. Only those participants with significant immunosuppression, those that had received blood products within 6 months of recruitment and those that had active and ongoing symptoms of SARS-CoV-2 infection (within the last 21 days) were excluded. Only samples from individuals with at least one positive test from any platform were included in the analysis. Moreover, individuals without a known symptom start date were removed.

### Data collection

As part of the Co-STARs study all participants undertook a detailed standardised online questionnaire at study entry^12^. This included the date of onset of COVID-19 symptoms, and any SARS-CoV-2 diagnostic test results.

### Comparison of serological assays

Samples taken as part of the Co-STARS study^12^ which had an accompanying symptom start date available for analysis were initially screened for seropositivity by the EDI assay or by any of the three antigens of the Meso Scale Discovery (MSD) assay. The selected samples each underwent testing with 4 serological assays: (1) The Roche Elecsys Anti-SARS-CoV-2 electrochemiluminescence immunoassay (ECLIA) assay detects the nucleocapsid (N) antigen (Roche-N); (2) the Roche Elecsys Anti-SARS-CoV-2 S electrochemiluminescence immunoassay (ECLIA) assay detects the spike (S) antigen (Roche-S); (3) the Abbott Nucleoprotein Chemiluminescent Microparticle Immunoassay (CLMIA) assay detects the nucleocapsid (N) antigen (Abbott-N); (4). All tests were performed as per manufacturer’s specifications.The four antigen Meso Scale Discovery (MSD) assay was undertaken at the WHO Pneumococcal Supranational Reference Laboratory at the UCL Institute of Child Health. Only 3 antigens were reported from the MSD assay (the Spike, the Nucleoprotein and the RBD) as the baseline test performance of the N-terminal domain (NTD) antibody response was insufficient for further evaluation as previously reported^13^. The Roche-N and Roche-S assays were undertaken by the Laboratory Medicine Service of Swansea Bay University Health Board, Morriston Hospital, Swansea. The Abbott-N assay was undertaken by Public Health Wales Microbiology at Cardiff and Vale University Hospital. All samples were stored and transported between laboratories at − 80 °C and only removed for aliquoting prior to testing to avoid unnecessary freeze–thaw cycles.

### Statistical analysis and modelling

In order to evaluate the relative proportion of seropositive tests in the parallel serological assays over time, a time-to-event analysis was performed using the time from symptom onset and the first negative test for each assay after a first positive test as the event of interest using the R package *survival*^14,15^. Only tests taken > 14 days after symptom onset were considered in the analysisNo tests were performed between 14- and 21-days post symptoms, and thus using 14 or 21 days post symptom onset as threshold did not affect our results. A participant was defined as seropositive when at least one of the 4 tests undertaken was seropositive. If the other tests that were run in parallel never became seropositive, the time-to-event was set to the earliest test taken for that individual. If a participant never became seronegative during the follow-up period, a right-censored observation was added at the time of the last serological test.

Additionally, the decay rate after 21 days since symptom onset was estimated using a Bayesian generalized linear mixed model as implemented in the R package *MCMCglmm*^16^, where time from symptom onset was included as a fixed effect and study participants as a random effect. Therefore, a unique slope for the regression was estimated for the entire population, while the intercept was allowed to vary between the study participants. The decay rate was estimated from the slope of the linear model.

To assess the overall diagnostic capability of the Abbott-N assay, a receiver operating characteristic curve (ROC) analysis was performed using the pROC package within R^17^. The MSD-N and Roche-N assays were used as the gold standard for the comparison.

### Ethical approval and consent

The study had national Integrated Research Application System (IRAS) approval and all participants in the study provided informed consent.

## Results

A total of 950 samples from 329 participants seropositive by any assay after 14 days underwent testing with the Roche-N, Roche-S, the MSD and the Abbott-N assay. The majority of the participants (98%, 321/329) had a positive result by two or more assays.

### Antibody decay with time

**Figure 1:**
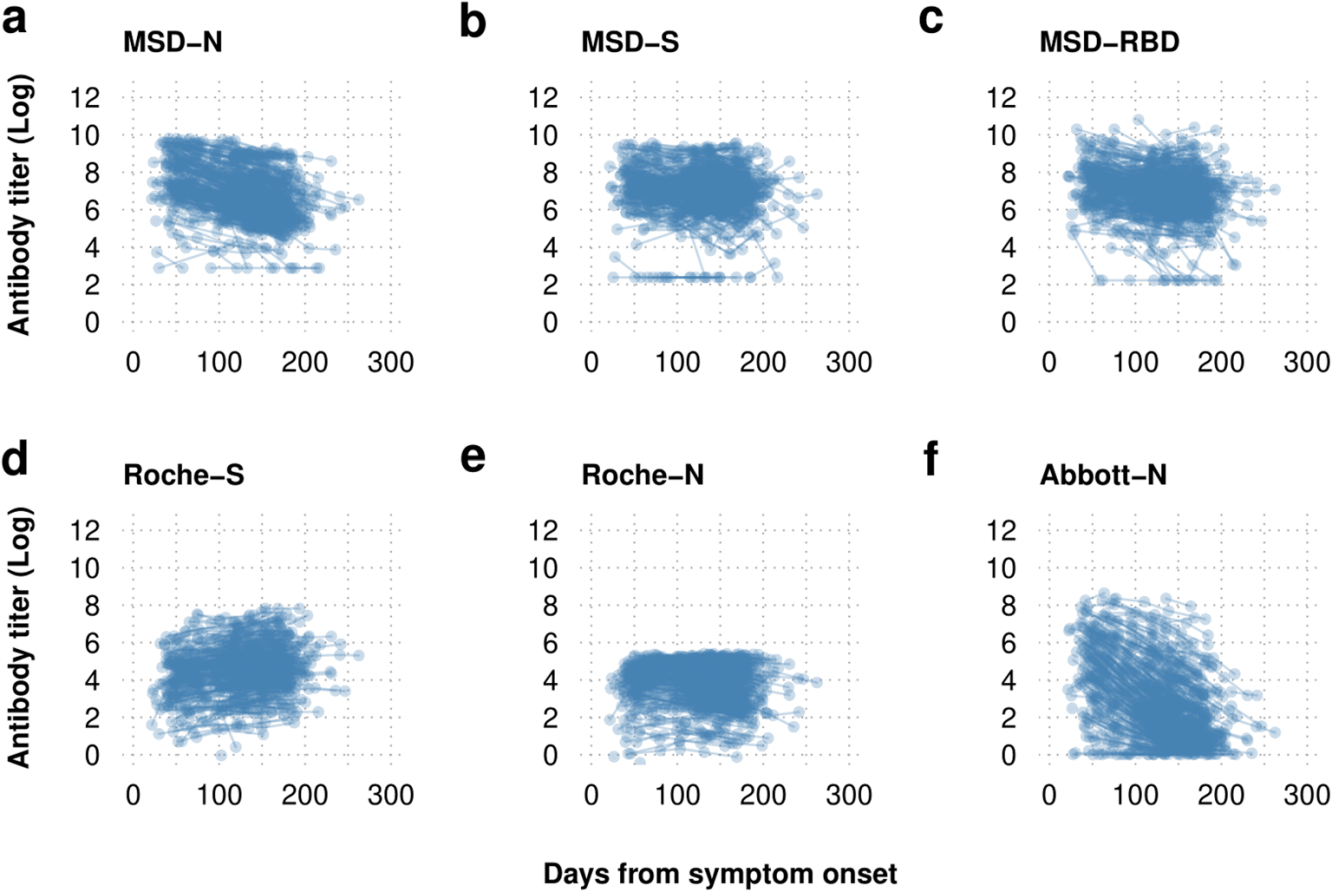
Log transformed serial serological antibody titer data plotted by time from symptom onset. Antibody dynamics are dependent on the assay used with the sensitive Roche-S and MSD-S assay demonstrating maintenance of the spike protein antibody while the nucleoprotein antibody is shown to wane with the MSD and Abbott-N assays but to a lesser extent with the Roche-N assay.

Plotting the raw log transformed antibody titers over time since symptom onset (Fig. 1) demonstrated that antibody dynamics were dependent on the assay undertaken. The production of spike antibodies was demonstrated to be maintained at high levels up to 200 days when evaluated by the MSD and the quantitative Roche -S assay. All nucleoprotein antibody assays demonstrated decay of the nucleoprotein antibody over time. This was most pronounced in the Abbott-N assay and much less so in the Roche -N assay which demonstrated slow waning of the nucleoprotein antibody.

### Assay sensitivity with time post symptom onset

**Figure 2:**
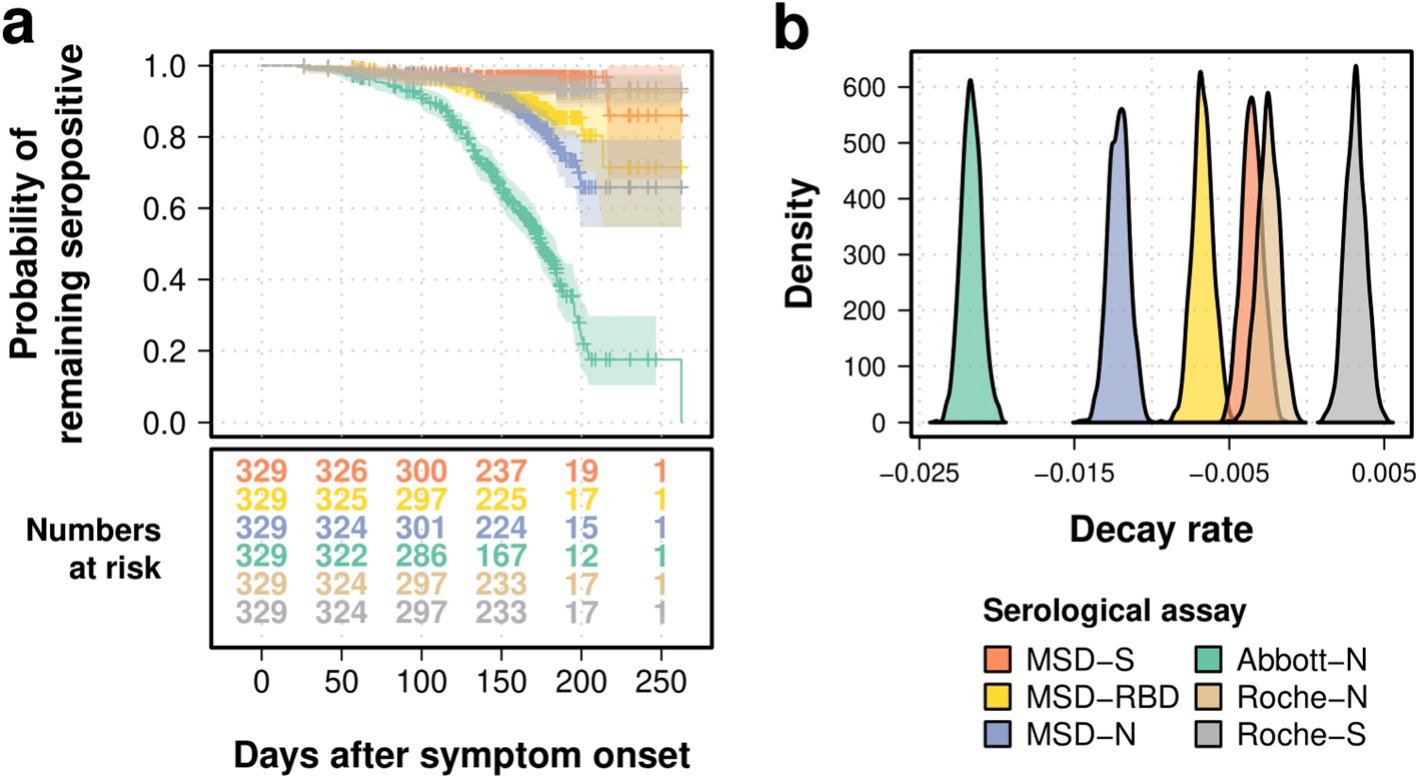
Comparison of seropositivity and antibody dynamics between serological tests. The Roche-S assay target the spike antibody, the Abbott-N and the Roche-N assays target the N-antibody while the MSD assay targets the N-, the S- and the antibody to the Receptor Binding Domain (RBD) of the spike protein in parallel. (**a**) Kaplan–Meier curve and numbers at risk (the number of participants under follow up with serological tests available for analysis at that time point) for different serological tests. Y-axis represents the probability of remaining seropositive, while the X-axis shows days after symptom onset with numbers of participants under follow up shown in the table below. (**b**) Inferred posterior density distributions of the decay rate in a generalized linear mixed model.

The existing published test performance for all assays undertaken is provided in Table 1. The sensitivity of all assays (at least 14 days from symptom onset) at 50, 100 and 150 days is provided in Table 2. All assays demonstrated a reasonable sensitivity at 50 days following infection (Fig. 2a). As time passed following infection, the Abbott-N assay rapidly became seronegative (Fig. 2a), with a median survival time inferred at 175 days (95% CI 168–185 days), whereas the survival probability at 150 days was inferred to be 95% for the Roche-N (95% CI 0.92–0.97), and 91% for the MSD-N assay (95% CI 0.87–0.94). The Roche-S and MSD-S assays remained seropositive for the duration of the study. The MSD-RBD assay showed some evidence of waning seropositivity over time (90% Survival probability at 150 days, 95% CI 0.88–0.94).

**Table 1:** A summary of the existing published data for the commercially available tests in this comparison.

Tests	Manufacturer	Target Antigen	Type	Sens^†^	Spec	# Samples/Patients*	Days post symptom onset
Abbott-N^18^	Abbott-N	Nucleoprotein	CLMIA	100.0% (day 17)	99.9%	689/125	≥ 21
Roche-N^19^	Roche Cobas	Nucleoprotein	ECLIA	99.5%	99.8%	496/102	≥ 14
Roche-S^20^	Roche Cobas	Spike protein	ECLIA	96.6%	100%	1485^‡^/331	≥ 15
MSD^13^	Meso Scale Discovery	Nucleoprotein	ECLIA	87.2%	92.8%	196/196	≥ 21
		Spike protein		97.9%	97.4%	47/47	≥ 21
		RBD (receptor binding domain)		93.6%	92.3%	47/47	≥ 21

The corresponding antigen target and published sensitivity, specificity, positive predictive value and negative predictive value (if available).

*ECLIA* Electro Chemiluminescent Immunoassay, *CLMIA* chemiluminescent microparticle immuno assay, *PPV* positive predictive value,* NPV* negative predictive value, *Sens* sensitivity, *Spec* specificity, *ELISA* enzyme linked immunosorbent assay.

^†^The highest reported sensitivity.

^‡^233 of these samples were tested at ≥ 15 days post PCR diagnosis.

*For sensitivity testing.

**Table 2:** Sensitivity of compared assays at 50, 100 and 150 days from symptom onset.

	Survival probability (95% CI)		
	50-day	100-day	150-day
Abbott-N	0.985 (0.97;0.99)	0.919 (0.89;0.95)	0.655 (0.6;0.71)
Roche-N	0.988 (0.98;1.0)	0.963 (0.94;0.98)	0.949 (0.92;0.97)
Roche-S	0.991 (0.98;1.0)	0.966 (0.95;0.99)	0.952 (0.93;0.98)
**MSD**			
N	0.988 (0.98;1.0)	0.972 (0.95;0.99)	0.907 (0.87;0.94)
S	0.997 (0.99;1.0)	0.978 (0.96;0.99)	0.968 (0.95;0.99)
RBD	0.994 (0.99;1.0)	0.969 (0.95;0.99)	0.909 (0.88;0.94)

A total of 45% (159/329) of the individuals had a negative result using the Abbott-N assay during the course of the study. For the MSD test, 16% (52/329) of participants had a negative test for the N antigen, 11% (36/329) for RBD, and 3% (11/329) for the S antigen. For the Roche platform, 5.5% (18/329) of the individuals had a negative result with the Roche-N assay, while only 4.8% (16/329) of them had a negative result for the S antigen over the course of the study.

### Mathematical model fits to estimate antibody decay

To estimate the decay rate for each antibody and assay studied, a generalized linear mixed model was fitted to the trajectory of antibody decay after 21 days from symptom onset, where the decay rate was estimated as the slope of the antibody titer through time. Under the most sensitive and quantitative Roche -S assay the spike antibody demonstrated no decay at all and rather a slow rate of increased titers over time from symptom onset (0.0031, 95% CI 0.0018–0.0044, Fig. 2b). In accordance with the raw observed data, all nucleoprotein antibodies under the mathematical model decayed. This was most pronounced in the Abbott-N assay (− 0.022, 95% CI − 0.023 to − 0.02) and least pronounced in the Roche -N assay (− 0.0025, 95% CI − 0.0039 to − 0.0012, Fig. 2b, Table 3).

**Table 3:** Decay rate for each serological assay (log arbitrary units per day) estimated in a generalized linear mixed model.

	Mean	95% CI
MSD-N	− 0.0121	− 0.0134; − 0.0107
MSD-RBD	− 0.0068	− 0.008; − 0.0055
MSD-S	− 0.0035	− 0.0048; − 0.0023
Abbott-N	− 0.0216	− 0.0229; − 0.0204
Roche-S	0.0031	0.0018;0.0044
Roche-N	− 0.0025	− 0.0039; − 0.0012

**Figure 3:**
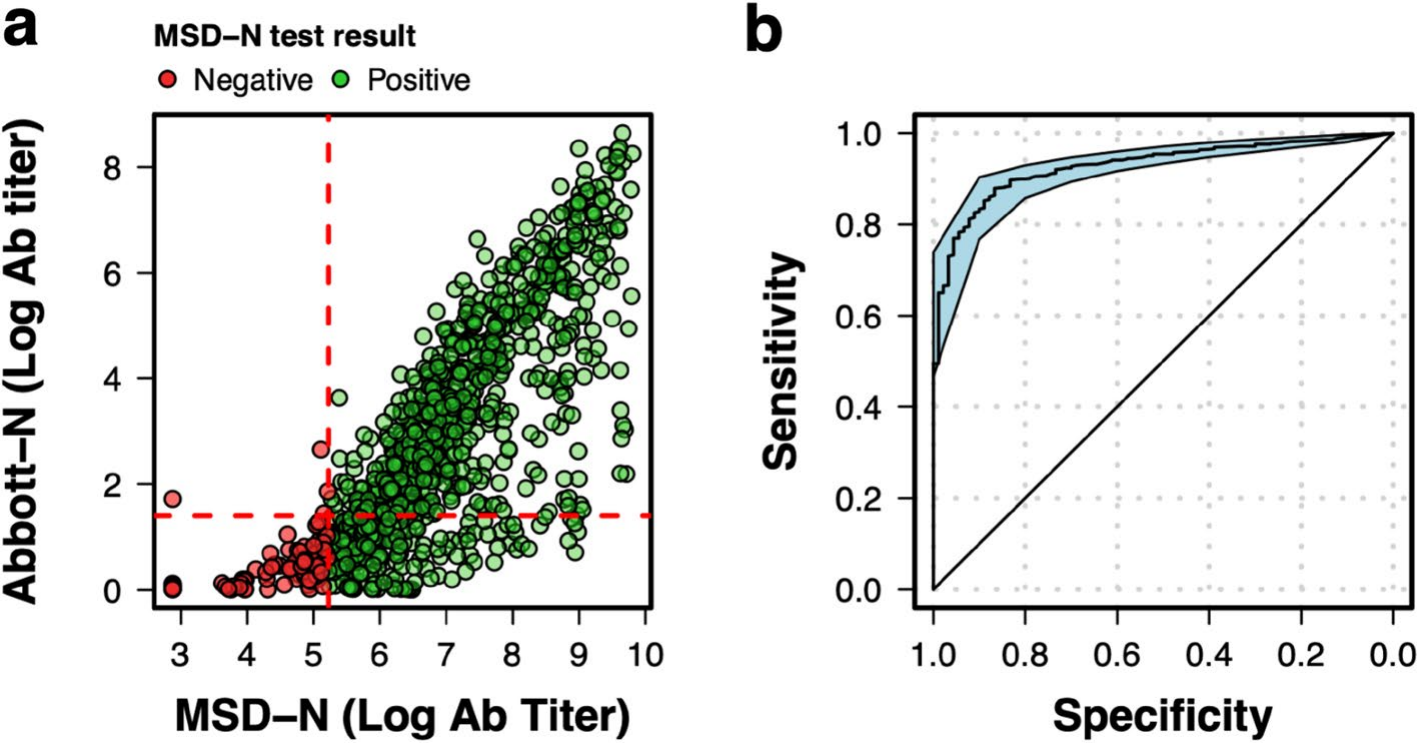
Comparison of antibody titers between the Abbott-N assay and the MSD-N assay. (**a**) The quantitative results for the MSD-N assay were compared to those of the Abbott-N test for each sample taken. Colours divide the samples depending on whether it was positive (green) or negative (red) for the MSD-N assay. Dotted red lines represent the seropositivity threshold for the Abbott-N assay (horizontal) and the MSD-N test (vertical). (**b**) ROC curve for the Abbott-N assay using the MSD-N test as gold standard. The x ~ y line represents the profile of a random classifier. Blue shaded area shows the 95% CI.

The lower performance of the Abbott-N assay can be explained by a lower detection of titer values as their concentration wanes over time. When compared to the quantitative MSD-N, 26% (222/860) of all positive samples by the MSD-N were negative for the Abbott-N test (Fig. 3a). A total of 75% of samples (137/ 183) positive by the MSD-N with an MSD arbitrary titer value lower than 403 were negative for the Abbott-N assay. Using the currently manufacturer recommended threshold of 1.4 arbitrary units, the Abbott-N test was characterized by a high specificity of 0.96 and a sensitivity of 0.74 using all our test results after 14 days. Using a ROC curve (Fig. 3b), the optimal cut-off that maximises both specificity and sensitivity was estimated to be 0.845 arbitrary units.

## Discussion

Sensitive measurement of SARS-CoV-2 seropositivity is key to evaluate who has been infected or exposed to SARS-CoV-2, to determine the correlates of protection from future disease, stratify those that need booster vaccination and target the use of anti-SARS-CoV-2 antibodies to those that are seronegative. To our knowledge no other study has evaluated the sensitivity of multiple diagnostic tests in parallel on longitudinally collected serological samples. This study demonstrates that as time elapses after infection, the sensitivity of serological testing varies widely depending on the test used. Although serological tests may be demonstrated to perform well 14–21 days after infection, this initial test performance often diminishes as time passes. In order to evaluate whether or not the population maintains SARS-CoV-2 antibodies it is vital that we utilize serological tests that remain sensitive over time.

Initial published baseline test performance reports concluded that the Abbott-N assay was a high-performance test and a key tool in SARS-CoV-2 surveillance^18^. Our data demonstrate that as time passes following infection the sensitivity of this assay declines rapidly until at < 6 months following infection it is no more than 50% sensitive. Our findings support the concerns raised by others regarding the poor performance of some nucleoprotein based assays^21,22^.

In contrast, the Roche assays, particularly the Roche Elecsys Anti-SARS-CoV-2 Spike assay maintained high sensitivity for the 200-day duration of the study. Although there remains no single correlate of sterilizing or protective immunity following SARS-CoV-2 infection or vaccination, it is clear that natural infection and the presence of neutralizing spike antibodies decreases the possibility of re-infection and the severity of disease upon re-exposure to currently circulating strains^23^. Our finding that spike antibodies remained at high titers 200 days after infection adds to our previous study on this topic^10^ and provides further evidence in support of long-lasting protection against severe disease from currently circulating strains. Fitting mathematical models to the raw data of the Roche spike assay demonstrated that spike antibody titers did not decay but rather increased slightly over the duration of the study. The Roche nucleoprotein assay also maintained sensitivity for the duration of the study with a low rate of decay. Although this assay is semi-quantitative, our findings suggest that this could be used to sensitively identify those that have been vaccinated from those that have been both vaccinated and infected.

Many studies have evaluated the impact of time on test sensitivity over the first 3 weeks following symptom onset^24–26^. However, we found no other study that had examined the sensitivity of antibody testing on parallel longitudinal samples collected between 1 and 6 months after infection or exposure. Assays with a higher titer cut-off for detection may perform well in the initial period after infection, but fail to detect seropositivity as antibody levels wane over time. We show that the Abbott-N test failed to detect 75% of samples positive for the MSD-N with a titer value lower than 403, which makes the Abbott-N assay less suitable for seroprevalence studies. Using a ROC curve and the MSD-N and Roche-N assays as the gold standard, we showed that a lower threshold of 0.845 instead of 1.4 arbitrary units may be more suitable to optimize the sensitivity and specificity. Even though different thresholds may be relevant depending on whether sensitivity or specificity needs to be prioritized, our findings suggest that the high Abbott-N test threshold results in a high number of false negatives. These findings are concordant with previous reports showing a range of high uncertainty between 0.49 and 1.4^27^. Barzin et al.^28^ used Abbott-N testing alone to determine SARS-CoV-2 seroprevalence in 2,973 asymptomatic out-patients in North Carolina estimating a seroprevalence of 0.8%. Similarly, Wilkins et al.^29^ used Abbott-N on 6510 healthcare workers up to 150 days after symptom onset and estimated a seroprevalence of 4.8%. Our findings suggest that previously published surveys of SARS-CoV-2 seroprevalence such as these could have significantly underestimated the true prevalence of SARS-CoV-2 humoral immunity.

Memory T-cell interferon gamma release or proliferation assays in response to SARS-CoV-2 antigens provide an alternative means of assessing prior exposure to infection. However, these assays are limited by cross reactive immunity to the seasonal coronaviruses decreasing specificity^30,31^.

Although all serological tests used in the study demonstrated a high initial specificity, one limitation is that only 38% of participants had a confirmatory SARS-CoV-2 PCR result. Our data may therefore be influenced by an unknown proportion of falsely positive serological tests. However, at entry to the study, all seropositive participants had both a screening EDI nucleoprotein assay and an MSD assay performed which limited the chances of a falsely positive result due to a single erroneous test. Not all samples were processed at the same time; the Roche and Abbott-N assays were processed 3 months after the MSD assays. Despite this, we believe that sample storage and freeze-thawing cycles are unlikely to have influenced our findings as the Roche quantitative spike assay was performed last and demonstrated the highest prolonged levels of spike antibody of all tests used.

In summary, although serological tests may demonstrate high sensitivity 3-weeks after SARS-CoV-2 infection, this is far from the case with some tests 6-months after infection. The Abbott-N assay performed poorly at this time, whereas the Roche and MSD tests maintained a high sensitivity for the 200 days of the study. Tests that perform poorly over time will lead to spurious estimates in population level seroprevalence studies and findings from these studies should be adjusted to account for sensitivity of the test used and the time since infection. Test performance as time passes post infection should be considered before evaluating who is a candidate for booster vaccination or anti-SARS-CoV-2 antibody therapy.

## Supplementary Information

Supplementary Information.

## Data Availability

The dataset used during the current study is available as Supplementary Data File 1.

## References

[1] Lisboa Bastos M, Tavaziva G, Abidi SK, Campbell JR, Haraoui L-P, Johnston JC (2020). Diagnostic accuracy of serological tests for covid-19: Systematic review and meta-analysis. BMJ.

[2] Kubina R, Dziedzic A (2020). Molecular and serological tests for COVID-19. A comparative review of SARS-CoV-2 coronavirus laboratory and point-of-care diagnostics. Diagnostics.

[3] la Marca A, Capuzzo M, Paglia T, Roli L, Trenti T, Nelson SM (2020). Testing for SARS-CoV-2 (COVID-19): A systematic review and clinical guide to molecular and serological in-vitro diagnostic assays. Reprod. Biomed. Online.

[4] Cheng MP, Yansouni CP, Basta NE, Desjardins M, Kanjilal S, Paquette K (2020). Serodiagnostics for severe acute respiratory syndrome-related coronavirus 2: A narrative review. Ann. Intern. Med..

[5] Public Health England. *Evaluation of the Abbott SARS-CoV-2 IgG for the Detection of Anti-SARSCoV-2 Antibodies*. https://assets.publishing.service.gov.uk/government/uploads/system/uploads/attachment_data/file/890566/Evaluation_of_Abbott_SARS_CoV_2_IgG_PHE.pdf (2020).

[6] Public Health England. *Evaluation of Roche Elecsys AntiSARS-CoV-2 Serology Assay for the Detection of Anti-SARS-CoV-2 Antibodies*. https://assets.publishing.service.gov.uk/government/uploads/system/uploads/attachment_data/file/891598/Evaluation_of_Roche_Elecsys_anti_SARS_CoV_2_PHE_200610_v8.1_FINAL.pdf (2020).

[7] Coste AT, Jaton K, Papadimitriou-Olivgeris M, Greub G, Croxatto A (2021). Comparison of SARS-CoV-2 serological tests with different antigen targets. J. Clin. Virol..

[8] Ainsworth M, Andersson M, Auckland K, Baillie JK, Barnes E, Beer S (2020). Performance characteristics of five immunoassays for SARS-CoV-2: A head-to-head benchmark comparison. Lancet Infect. Dis.

[9] Pollán M, Pérez-Gómez B, Pastor-Barriuso R, Oteo J, Hernán MA, Pérez-Olmeda M (2020). Prevalence of SARS-CoV-2 in Spain (ENE-COVID): A nationwide, population-based seroepidemiological study. The Lancet.

[10] Grandjean L, Saso A, Torres Ortiz A, Lam T, Hatcher J, Thistlethwayte R (2021). Long-term persistence of spike protein antibody and predictive modeling of antibody dynamics after infection with severe acute respiratory syndrome coronavirus 2. Clin. Infect. Dis..

[11] Bauer G, Struck F, Schreiner P, Staschik E, Soutschek E, Motz M (2021). The challenge of avidity determination in SARS-CoV-2 serology. J. Med. Virol..

[12] Great Ormond Street Hospital for Children NHS Foundation *Trust. COVID-19 Staff Testing of Antibody Responses Study (CO-STARS)*. https://clinicaltrials.gov/ct2/show/NCT04380896 (2021).

[13] Johnson M, Wagstaffe HR, Gilmour KC, Mai AL, Lewis J, Hunt A (2020). Evaluation of a novel multiplexed assay for determining IgG levels and functional activity to SARS-CoV-2. J. Clin. Virol..

[14] R Core Team (2018). R: A Language and Environment for Statistical Computing.

[15] Therneau TM, Grambsch PM (2000). Modeling Survival Data: Extending the Cox Model.

[16] Hadfield JD (2010). MCMC methods for multi-response generalized linear mixed models: The MCMCglmm R package. J. Stat. Softw..

[17] Robin X, Turck N, Hainard A, Tiberti N, Lisacek F, Sanchez J, Müller M (2011). pROC: An open-source package for R and S+ to analyze and compare ROC curves. BMC Bioinform..

[18] Bryan A, Pepper G, Wener MH, Fink SL, Morishima C, Chaudhary A (2020). Performance characteristics of the abbott architect sars-cov-2 igg assay and seroprevalence in Boise, Idaho. J. Clin. Microbiol..

[19] Muench P, Jochum S, Wenderoth V, Ofenloch-Haehnle B, Hombach M, Strobl M (2020). Development and validation of the Elecsys anti-SARS-CoV-2 immunoassay as a highly specific tool for determining past exposure to SARS-CoV-2. J. Clin. Microbiol..

[20] FDA. Elecsys Anti-SARS-CoV-2 S - Instructions for Use 2021. https://www.fda.gov/media/144037.

[21] Rosadas C, Randell P, Khan M, McClure MO, Tedder RS (2020). Testing for responses to the wrong SARS-CoV-2 antigen?. The Lancet.

[22] Bolotin S, Tran V, Osman S, Brown KA, Buchan SA, Joh E (2021). SARS-CoV-2 seroprevalence survey estimates are affected by anti-nucleocapsid antibody decline. J. Infect. Dis..

[23] Hall VJ, Foulkes S, Charlett A, Atti A, Monk EJM, Simmons R (2021). SARS-CoV-2 infection rates of antibody-positive compared with antibody-negative health-care workers in England: A large, multicentre, prospective cohort study (SIREN). The Lancet.

[24] Whitman JD, Hiatt J, Mowery CT, Shy BR, Yu R, Yamamoto TN (2020). Evaluation of SARS-CoV-2 serology assays reveals a range of test performance. Nat. Biotechnol..

[25] Piec I, English E, Thomas MA, Dervisevic S, Fraser WD, John WG (2021). Performance of SARS-CoV-2 serology tests: Are they good enough?. PLoS ONE.

[26] Wang H, Ai J, Loeffelholz MJ, Tang YW, Zhang W (2020). Meta-analysis of diagnostic performance of serology tests for COVID-19: Impact of assay design and post-symptom-onset intervals. Emerg. Microbes Infect..

[27] Castro MDM (2021). Performance verification of the Abbott SARS-CoV-2 test for qualitative detection of IgG in Cali, Colombia. PLoS ONE.

[28] Barzin A, Schmitz JL, Rosin S, Sirpal R, Almond M, Robinette C (2020). SARS-CoV-2 seroprevalence among a Southern U.S. population indicates limited asymptomatic spread under physical distancing measures. MBio.

[29] Wilkins JT, Gray EL, Wallia A, Hirschhorn LR, Zembower TR, Ho J (2021). Seroprevalence and correlates of SARS-CoV-2 antibodies in health care workers in Chicago. Open Forum Infect. Dis..

[30] le Bert N, Tan AT, Kunasegaran K, Tham CYL, Hafezi M, Chia A (2020). SARS-CoV-2-specific T cell immunity in cases of COVID-19 and SARS, and uninfected controls. Nature.

[31] Lipsitch M, Grad YH, Sette A, Crotty S (2020). Cross-reactive memory T cells and herd immunity to SARS-CoV-2. Nat. Rev. Immunol..

